# The path from the choroid plexus to the subventricular zone: go with the flow!

**DOI:** 10.3389/fncel.2012.00034

**Published:** 2012-08-09

**Authors:** Ana Mendanha Falcão, Fernanda Marques, Ashley Novais, Nuno Sousa, Joana A. Palha, João Carlos Sousa

**Affiliations:** ^1^School of Health Sciences, Life and Health Sciences Research Institute (ICVS), University of MinhoBraga, Portugal; ^2^ICVS/3B's—PT Government Associate LaboratoryBraga/Guimarães, Portugal

**Keywords:** choroid plexus, cerebrospinal fluid, subventricular zone, growth factors

## Abstract

In adult mammals, under physiological conditions, neurogenesis, the process of generating new functional neurons from precursor cells, occurs mainly in two brain areas: the subgranular zone in the dentate gyrus of the hippocampus, and the subventricular zone (SVZ) lining the walls of the brain lateral ventricles. Taking into account the location of the SVZ and the cytoarchitecture of this periventricular neural progenitor cell niche, namely the fact that the slow dividing primary progenitor cells (type B cells) of the SVZ extend an apical primary cilium toward the brain ventricular space which is filled with cerebrospinal fluid (CSF), it becomes likely that the composition of the CSF can modulate both self-renewal, proliferation and differentiation of SVZ neural stem cells. The major site of CSF synthesis is the choroid plexus (CP); quite surprisingly, however, it is still largely unknown the contribution of molecules specifically secreted by the adult CP as modulators of the SVZ adult neurogenesis. This is even more relevant in light of recent evidence showing the ability of the CP to adapt its transcriptome and secretome to various physiologic and pathologic stimuli. By giving particular emphasizes to growth factors and axonal guidance molecules we will illustrate how CP-born molecules might play an important role in the SVZ niche cell population dynamics.

## Introduction

The adult subventricular zone (SVZ) neural stem cell niche, also designated as subependymal zone to distinguish from the embryonic SVZ, is the major source of novel neurons in the adult brain (Whitman and Greer, 2009). The properties of this neural progenitor cells niche are being increasingly studied, in light of the potential usage of endogenous sources of regenerative cells in disorders of the central nervous system. The adult SVZ stem cell population is heterogeneous, in a region-specific manner, along the wall of the brain ventricles. This stem cells heterogeneity is a consequence of the pattern of transcription factors (intrinsic factors) they express (Alvarez-Buylla et al., 2008), and results in the generation of different types of novel neurons in the olfactory bulb (Lledo et al., 2008). In addition several extrinsic factors [other brain cells, blood vessels, and the cerebrospinal fluid (CSF)] in the vicinity of the SVZ also participate in the regulation of the SVZ niche and in fate determination of these progenitor cells. The CSF, whose composition is mainly determined by the choroid plexus (CP) secretome, is a major source of proteins and smaller molecules that signal the SVZ. Understanding the contribution of the CP in the interplay between extrinsic factors and intrinsic properties of the SVZ neural progenitor cells is not only of biological relevance, but also of interest in pathological conditions that may alter the CP transcriptome and/or secretome, and ultimately impact on the SVZ.

## The CP morphology and function

**Figure 1:**
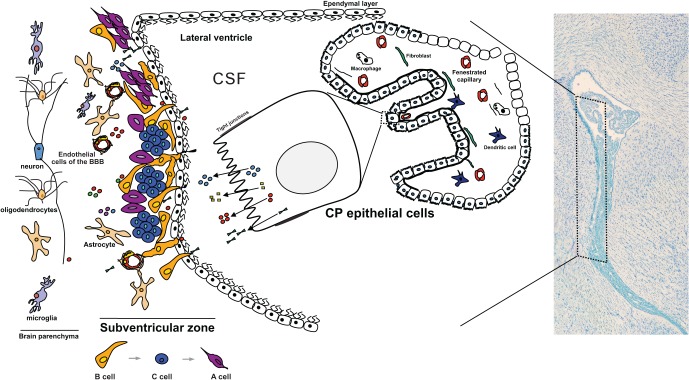
**The CP influences the CSF composition that baths the neural progenitor cells in the SVZ.** Due to its highly secretory capacity and its particular location facing the lateral wall of the brain ventricles, and hence the SVZ, the proteins being secreted by the CP rapidly flow in the CSF and enter in contact with the SVZ. Proteins and other molecules that are secreted toward the CSF penetrate the interstitial space between the cells adjacent to the wall of the ventricles. Being composed of a thin cell layer, the SVZ is influenced by the paracrine effect of the CP. In particular, type B1 neural stem cells are in direct contact with the CSF by projecting a primary cilium toward the ventricle. These cells are considered the stem cells of the adult SVZ and give rise to type C cells (transit amplifying progenitors) that in turn originate the type A cells (neuroblasts). Also in the SVZ are the resident astrocytes, microglia and endothelial cells of the blood-brain barrier (BBB). Together with the ependymal cells and the CP-born molecules, these are the modulators of the adult SVZ cell niche. Cells of the brain parenchyma lay in the SVZ neighborhood.

The CPs are thin membranes that float in the CSF filled lateral, third and fourth brain ventricles. The CP is mainly composed of a monolayer of epithelial cells derived from the ependymal cells that line the wall of the brain ventricles (Figure 1). Underneath this monolayer of epithelial cells lays a stroma perfused with highly permeable fenestrated blood vessels, fibroblasts, and immune cells such as dendritic cells and macrophages. The CP is a highly vascularized structure with a 10 times fold higher blood flow when compared to the brain parenchyma (Keep and Jones, 1990).

CP epithelial cells display a clearly polarized cellular morphology bearing: (1) an apical surface (facing the brain ventricles, and hence the CSF) composed of a large number of microvilli of variable length that extensively increases the contact area with the CSF; (2) a smother basolateral membrane (facing the CP connective tissue, hence the blood side); and (3) lateral membranes, the surface contact area between adjacent epithelial cells. At the most apical portion of the lateral membranes the existence of tight junctions limits the paracellular passage of blood derived cells and proteins (Vorbrodt and Dobrogowska, 2003). Tight junctions, together with the expression of several basolateral and apical transporters, make CP epithelial cells the effectors of the blood-CP-CSF barrier (Spector, 2010). The CP is responsible for the generation of at least two-thirds of the CSF volume via the secretion of water, ions, and macromolecules (Johanson et al., 2008). In fact, the CP epithelial cells display several transporters for water molecules and ions, transporters for small peptides and polypeptides, and have the capacity to synthetize, and then secrete several proteins toward the CSF (Praetorius, 2007; Johanson et al., 2008). The necessary energy to feed this highly secretory capacity is provided by a high density of mitochondria (Redzic and Segal, 2004). In addition, the CP epithelial cells have receptors, both in the apical and basolateral sides, for molecules such as neurotransmitters, cytokines, bacterial toxins, amongst others; importantly, several of these receptors have been shown to signal downstream cascades that ultimately influence the CP transcriptome and secretome (Marques et al., 2009a, 2011; Johanson et al., 2011).

## Proteins that are expressed by the CP are secreted toward the CSF

The high secretory capacity of the CP is reflected in the composition of the CSF (Chodobski and Szmydynger-Chodobska, 2001; Thouvenot et al., 2006) (Figure 1). Amongst the most abundant proteins in CSF are CP-secreted proteins, such as transthyretin (Sousa et al., 2007), transferrin and prostaglandin D2 synthase (Chodobski and Szmydynger-Chodobska, 2001). Reflecting the importance of the CP-CSF nexus in the normal brain functioning, these and other proteins have been independently explored as unique biomarkers of psychiatric and neurological disorders.

In the last decade, the continuous improvement of large screening proteomic techniques resulted in a more comprehensive characterization of the CSF protein content in several species and different ages (Parada et al., 2006; Zappaterra et al., 2007; Stoop et al., 2010), both in physiological and in neuropathological conditions such as Alzheimer's disease and depression (Ditzen et al., 2011; Menon et al., 2011; Ringman et al., 2012). However, changes in the CSF content may result not only from alteration in the CP, but also (or rather) be the consequence from an altered brain parenchyma metabolism under the pathological condition.

## The SVZ stem cell niche is in close contact with the CSF

The adult SVZ niche is located along the lateral walls of the lateral brain ventricles (Figure 1). It is composed of slow-dividing (type B) and fast-dividing (type C) stem cells, and neuroblasts (type A cells) (García-Verdugo et al., 1998). The slow-dividing stem cells are divided in two distinct types, B1 and B2 cells, based on cellular characteristics and positioning in the SVZ. Type B1 cells are located closer to the ventricular space, with cell bodies immediately below the layer of ependymal cells, and are in direct contact with the CSF by a unique short non-motile primary cilium that extends toward the ventricles (Mirzadeh et al., 2008). In addition, type B1 cells are also in contact, by means of basal processes, with the extensive network of blood vessels that cross the periventricular space (Shen et al., 2008). By means of gap and adherens junctions, type B1 cells also contact each other and ependyma cells (Mirzadeh et al., 2008). Type B2 stem cells have astrocytic characteristics and are not in direct contact with the CSF, but rather located closer to the striatum. Type C cells, also known as transit-amplifying cells (TAPs), are the direct progeny of type B cells and are in close contact with their progenitors and with the vasculature. These fast-proliferating cells originate neuroblasts (type A cells) that migrate along the rostral migratory stream (RMS) anteriorly toward the olfactory bulbs where they differentiate mainly into GABAergic interneurons (Whitman and Greer, 2009). The astrocytic processes together with the blood vessels form a scaffold that directs these chains of migrating neuroblasts toward the olfactory bulbs (Whitman and Greer, 2009; Bozoyan et al., 2012). The SVZ also gives rise to oligodendrocyte progenitors, although in lower number when compared with neuroblasts (Menn et al., 2006).

The cells in the SVZ are influenced by several types of extrinsic factors namely growth factors, neurotransmitters and other effectors of signaling pathways. These extrinsic factors originate from blood vessels (Shen et al., 2008), the ependymal cell layer (Lim et al., 2000), the nervous projections toward the lateral walls of the brain ventricles (Lennington et al., 2011), and most importantly, from the CSF. In fact, the distinctive architecture of the adult SVZ neural stem cell niche makes the CSF an essential player that influences the dynamics of the SVZ cell niche. This CSF born molecules influence, by direct contact, type B1 cells via the primary cilium and ependyma cells that in turn interact with type B1 cells. Moreover they also diffuse into the lateral wall parenchyma and thus can exert an effect in type B2, type C and type A cells.

## CP born molecules modulate the SVZ

The relevance of the CSF content during brain development has been extensively reported. For instance, CSF insulin-growth factor 2 (IGF2), is well described to promote growth and neuronal survival in the mouse developing cortex (Lehtinen et al., 2011). Another example is retinoic acid (Parada et al., 2008; Lehtinen et al., 2011); both meningeal- and CP-derived retinoic acid signaling were shown to contribute to cortical neuron formation and migration, and to cerebellum development (Zhang et al., 2003; Siegenthaler et al., 2009; Crandall et al., 2011).

While the relevance of CSF-derived molecules for neurogenesis during brain development has been evidenced, the potential of CSF-derived molecules to determine neural stem cells renewal, proliferation and migration in the postnatal neurogenic niches, namely the SVZ, has not received the same attention. Moreover, the specific contribution from CP secreted proteins has been seldom highlighted and only rarely demonstrated (Sawamoto et al., 2006). We will next refer to molecules that have been shown to influence the SVZ neural stem cells population dynamics, and that also are expressed/secreted by the adult CP.

### Insulin-like growth factors

IGF2 is highly expressed, as shown by in situ hybridization, in CP epithelial cells not only during development (Lehtinen et al., 2011) but also in adulthood (Bondy et al., 1992). Under physiological conditions IGF2 was the second highest expressed gene found in a microarray study of the adult CP (Marques et al., 2011); the other member of the insulin growth factor family, insulin-like growth factor 1 (IGF1), is also expressed by adult CP epithelial cells, but only modestly. While the CP is not the only contributor to the presence of IGF1 and IGF2 in the CSF during development (other sites are the meninges and the endothelial cells of the brain blood vessels) (Lehtinen et al., 2011) it is certainly well positioned to rapidly influence the SVZ by paracrine effects. Both IGF1 and IGF2 proliferative actions are signaled via the insulin-like growth factor type 1 receptor (IGFR1) (Weber et al., 1992). IGF2 was found to be highly associated with the primary cilium of cortical progenitor cells that projects directly toward the CSF, indicating that IGF signaling occurs via IGF1R located in the primary cilia (Lehtinen et al., 2011). In fact, ablation of IGFR1 expression solely in neural precursor cells resulted in impaired cortical formation, namely microcephaly (Kappeler et al., 2008), similarly to what was found in IGF2-null mice (Lehtinen et al., 2011). Noticeably, IGFR1 is present both in the apical portion of the developing cortical ventricular zone surface, and in the adult SVZ (Yan et al., 2006), which highlights the importance of CP-CSF derived IGF signaling. In turn, IGF1's role in adult neurogenesis has been extensively demonstrated for the dentate gyrus since IGF1 infusion into the hippocampus increases proliferation and neurogenesis (Anderson et al., 2002). Similarly, when infused into the ventricles, IGF1 promotes cell proliferation and neurogenesis in the adult hypothalamus (Pérez-Martín et al., 2010). Furthermore, IGF1 seems to promote the exit of neuroblasts from the adult SVZ and their migration toward the olfactory bulb (Hurtado-Chong et al., 2009). This mismatch in the role of IGF1 in the hippocampus and adult SVZ niches results not only from the differences in the cellular distribution pattern of IGFR1 in both regions (Anderson et al., 2002), but also from the interaction of the IGF1 signaling cascade with other signaling factors such as BDNF and VEGF (Llorens-Martín et al., 2009). Noteworthy is the fact that the action of IGF1 in the two principal adult niches might also be conditioned by its binding to insulin growth factor binding proteins that might inhibit or potentiate the action of IGF1.

Of interest, the adult CP also expresses (Marques et al., 2011) and secretes (Thouvenot et al., 2006) several other insulin growth factor related proteins, such as insulin growth factor binding proteins, insulin growth factor receptors and insulin growth factor binding protein-like 1 (Igfbpl1). Of notice, the latter, is a protein found enriched in adult neural stem cells when compared with parenchymal astrocytes obtained by fluorescence-activated cell sorting (FACS) of SVZ cells (Beckervordersandforth et al., 2010). As for insulin-like growth factor binding protein 2 (Igfbp2), which is very highly expressed by the adult CP (Marques et al., 2011), its ability to support the survival and cycling of hematopoietic stem cells has been recently shown (Huynh et al., 2011). Whether IGFBP2 has a similar effect in the neural progenitor cell population in the adult SVZ is still unknown.

### Fibroblast growth factors

Another important group of growth factors expressed by the adult CP (Marques et al., 2011) is the fibroblast growth factor (FGF) family and related proteins. Their involvement in several processes of brain development (neural stem cell induction, cell differentiation, brain regions patterning, and neuronal circuit assembly) has been extensively demonstrated (Guillemot and Zimmer, 2011). In fact, the embryonic brain has several sources of different FGF family members that contribute to the determination of brain regionalization; for example, the contribution of CSF-derived FGFs to embryonic brain development was shown for FGF2 since it promotes precursor proliferation in chick embryos (Martín et al., 2006). FGF8, potentially derived from the CP-CSF, has also been described to participate in the patterning of brain regions in the chick (Parada et al., 2005) and mouse embryos (Fukuchi-Shimogori and Grove, 2001).

As for the adult brain, FGF2 injected in the ventricles increased proliferation in the SVZ and neurogenesis in the olfactory bulb (Kuhn et al., 1997). Furthermore subcutaneously injected FGF2, in both early post-natal and in young rats, crossed the brain barriers and increased in the CSF while promoting proliferation in both the subgranular zone of the dentate gyrus and the SVZ (Wagner et al., 1999). The importance of FGF2 for neuronal proliferation is illustrated by the extensive use of FGF2 (also known as bFGF) as a mitogen in SVZ neurosphere assays (Pastrana et al., 2011).

While less is known for the role of other FGF family members in adult neurogenesis it is of notice that FGF family members, such as FGF3, FGF9 and FGF10, are expressed under basal physiological conditions in the adult CP (Marques et al., 2011). Interestingly, FGF10 participation in the maintenance of the neurogenic potential of the adult SVZ was already suggested (Hajihosseini et al., 2008). Thus, under particular conditions, alterations in the expression of FGFs and their secretion toward the CSF may impact in the SVZ population.

### Epidermal growth factor (EGF) and transforming growth factor alpha (TGFa)

In vitro, when exposed to epidermal growth factor (EGF), adult SVZ derived cells form neurospheres that display multipotent and self-renewal properties (Pastrana et al., 2011). Although expressed in a relative small number of type B1 cells, EGF receptor (EGFR) expressing cells that form neurospheres in vitro are derived mainly from transit amplifying C cells (Doetsch et al., 2002). *In vivo*, it was shown that high levels of EGF administered by intracerebroventricular infusion impacts on the SVZ by increasing proliferation and generating progeny that occupies the surrounding brain parenchyma, and also diverts SVZ cells from the neuronal lineage to the oligodendrocytic lineage (Doetsch et al., 2002; Gonzalez-Perez et al., 2009). While it is believed (Doetsch et al., 2002) that the probable source of EGFR signaling occurs via transforming growth factor alpha (TGFa), given its expression in the CP (Seroogy et al., 1993; Marques et al., 2011), we cannot exclude that this signaling pathway occurs through EGF, also expressed by the CP (Marques et al., 2011). The relevance of TGFa/EGF for EGFR in this context is illustrated by the observation that the decreased proliferation in the SVZ displayed by the TGFa knockout mice can be corrected by supplementation with EGF (Tropepe et al., 1997). Interestingly, TGFa signaling via EGFR was also shown to influence the migratory properties of cells in the RMS (Kim et al., 2009) and of oligodendrocyte precursors derived from SVZ cells (Gonzalez-Perez and Quiñones-Hinojosa, 2010). In addition, a role for TGFa/EGFR signaling in promoting migration of cells derived from the SVZ was highlighted by TGFa infusion to the dopamine-depleted striatum of rodent models of Parkinson's disease (Cooper and Isacson, 2004; de Chevigny et al., 2008). Despite the interesting potential of the CP as a source of TGFa/EGF for the modulation of the SVZ its specific physiological contribution has never been demonstrated, which certainly deserves additional research.

### Platelet derived growth factors (PDGF)

PDGF signaling also occurs via the primary cilium and, interestingly, the PDGF signaling pathway modulates neural stem cells and affects lineage fate. For instance, *in vitro* experiments showed that PDGF increased neurosphere formation (Jackson et al., 2006). Whether SVZ GFAP-positive neural stem cells express the platelet derived growth factor receptor alpha polypeptide (PDGFRa) is disputable (Jackson et al., 2006; Chojnacki et al., 2011; Ihrie and Álvarez-Buylla, 2011), but infusion of PDGF into the ventricle is known to bolster proliferation in the SVZ (Jackson et al., 2006). The endogenous source of this ligand has not been determined (Ihrie and Álvarez-Buylla, 2011) but recently we found that the CP expresses several PDGFs mRNAs (Marques et al., 2011), with particular emphasis to PDGFa. Once again, the physiological significance of this expression and its influence over the SVZ neural stem cell niche remains to be established.

### Bone morphogenetic proteins (BMPs), sonic hedgehog (Shh) and Wnt

During the formation of the central nervous system, FGF signaling action is frequently aligned with and/or counteracted by signaling from BMPs, Shh and Wnt pathways. The result of this interaction, impacting on cell proliferation and cell fate, is dependent of the highly dynamic spatiotemporal variation in the expression of the various effector proteins (Guillemot and Zimmer, 2011).

The role of BMP, Shh, and Wnt proteins derived from the CP-CSF during development has been shown. For instance, Shh expression in the hindbrain CP is high and CSF Shh was demonstrated to be essential for cerebellar development by promoting proliferation of granule precursors (Huang et al., 2009, 2010). BMPs, that together with Wnt and FGF proteins, participate in cortical development (Shimogori et al., 2004), display a very dynamic presence in the CSF in an age dependent manner (Lehtinen et al., 2011).

In the adult SVZ, all these proteins participate in the regulation of the SVZ niche. Adult SVZ type B and C cells express both BMP2 and BMP4 and their respective receptors, and SVZ ependymal cells alter the activity of BMPs (Lim et al., 2000; Peretto et al., 2004). The CP origin of these ligands should also be considered since it expresses BMP1, BMP2, BMP4, BMP6 and BMP7 under basal physiological conditions (Marques et al., 2011). Noteworthy, the presence of some of these BMPs in the adult CSF was also recently confirmed (Lehtinen et al., 2011). Furthermore, growth differentiation factors 3 and 8 (Gdf3 and Gdf8), known to modulate BMP signaling, are also expressed by the adult CP (Marques et al., 2011) and are secreted toward the CSF (Lehtinen et al., 2011).

Shh and Wnts pathways are also active in the adult SVZ and were implicated in the formation, maintenance, proliferation and migration of adult neural stem cells. Shh is produced by ventral forebrain neurons that extend projections toward the SVZ, and persistent Shh signaling determines a specific neural progeny (Ihrie et al., 2011). Shh is also expressed by the CP (Marques et al., 2011) but its levels in the adult CSF have not been determined. As for Wnt, evidence exists that it may participate in the regulation of the adult SVZ population, namely enhancing Wnt signaling via beta-catenin in type C cells increases proliferation and results in a higher number of neurons in the olfactory bulb (Adachi et al., 2007). On the other hand, during embryonic development Wnt may rather play a role in maintaining the SVZ stem cell pool (Piccin and Morshead, 2011). Similarly to Shh and BMPs, several members (for instance Wnt5a, 5b, and 10b) of the Wnt family are expressed in the adult CP, and specifically secreted by CP epithelial cells, under basal conditions (Thouvenot et al., 2006; Marques et al., 2011).

One of the most interesting features of the Shh and Wnt signaling pathways is that they are modulated by the primary cilium (Louvi and Grove, 2011), making the cilia projected by type B1 cells toward the CSF a particularly well positioned route for CP derived “messages”.

### Ephrins, semaphorins and SLITs

Neuroblasts derived from the SVZ form a stream of moving cells that converge in the RMS; they are ensheated by a layer of astrocyte processes and use each other as guides in the migration process toward the olfactory bulb (Lledo et al., 2008). Several proteins from the group of classical axon guidance molecules were implicated not only in the regulation of the migration of neuroblasts, but also in the proliferation of type B cells. For instance, SVZ cells express the EphA and EphB receptors; the ligands for these receptors are the transmembrane molecules ephrins. The relevance of these molecules in the modulation of the SVZ was shown by the infusion of the EphB2 ligand in the lateral ventricles, which disrupted the migratory chain of neuroblasts and increased the proliferation of type B1 cells (Conover et al., 2000). Interestingly, a role for EphB2 signaling was suggested in the conversion of ependymal cells to astrocytes after lesion of the ventricular wall (Nomura et al., 2010). Also, it has recently been shown that ephrinB3-EphB3 signaling in the SVZ is transiently inhibited to allow the expansion and survival of neural progenitor cells upon traumatic brain injury (Theus et al., 2010). EphrinB3 is one of the ephrins expressed by the CP at very low levels under basal physiological conditions (Marques et al., 2011). While the fact that these molecules are attached to the membrane might diminish the relevance of their expression in the CP in the modulation of the SVZ neural progenitors, the data provided by infusion experiments (Conover et al., 2000) suggest that the cleavage and release of ephrins from the membrane of CP cells toward the CSF, under particular conditions, could influence the response of type B1 cells.

Semaphorins and slits are guidance molecules that, contrary to ephrins, are secreted factors, and hence exert their effects to a certain distance, in a paracrine fashion. Several semaphorin family members and slits 1, 2, and 3 are expressed (Marques et al., 2011) and secreted by the CP (Hu, 1999; Sawamoto et al., 2006). Semaphorin signaling is usually associated with endothelial cells (Tamagnone and Mazzone, 2011); semaphorin-3a and its respective receptor are highly expressed in the endothelial cells that are present along the RMS (Meléndez-Herrera et al., 2008), suggesting a role for semaphorin-3a in the migration of neuroblasts. Whether semaphorins specifically secreted by the CP influence the migration of neuroblasts toward the olfactory bulb has not been determined.

On the other hand, a role for slits specifically derived from the CP in the modulation of SVZ neural progenitors niche was clearly shown; for instance, slit 2 was shown to participate in the regulation of neuronal migration of neurons in the developing brain (Hu, 1999). Interestingly both the adult SVZ and the RMS express the receptors Robo2 and Robo3 through which slit1 and slit 2 exert their chemorepulsive activities (Nguyen-Ba-Charvet, 2004). Importantly, it was shown (Sawamoto et al., 2006) that ciliary beating from ependymal cells that line the wall of the brain ventricles contribute to the movement of the CSF, hence creating gradients of the chemorepelent slits secreted by the CP (Nguyen-Ba-Charvet, 2004; Marques et al., 2011) and thus contributing to the anterior migration of neuroblasts to the olfactory bulbs. Nevertheless, the role of slits in directing the migration of future olfactory bulb neurons goes behind the influence of the CP since type C and type A cells also express slit1 (Nguyen-Ba-Charvet, 2004), which seems to avoid the invasion of the RMS by astrocytic processes (Kaneko et al., 2010).

## Pathological conditions can alter the CP transcriptome/secretome and impact the SVZ

Being placed at the interface between the periphery and the central nervous system, the CP is particularly well positioned to sense alterations, and respond to, in both its basolateral side (the blood side) and its apical interface (the CNS side). On the other hand, any response the CP mounts to external stimuli will ultimately reflect in its secretome, and hence in the CSF that surrounds the brain parenchyma. In fact, since the CP epithelial cells are equipped with transporters for several proteins and metabolites, pathological damage to the CP itself will alter CSF composition and ventricular volume, as in the case of hydrocephalus (Johanson et al., 2011).

One relevant example of a peripheral event that impacts in the CP transcriptome is peripheral inflammation. When a single intraperitoneal injection of lipopolysaccharide, the membrane component of gram negative bacteria, was given, the CP displayed an acute and transient but profound change in its transcriptome that reflected in the CSF composition (Marques et al., 2007, 2009a). When a similar but repeated inflammatory stimulus was given, the result was still present but more attenuated (Marques et al., 2009b). These alterations in the CP properties and secretome may impact, even if transiently, in the SVZ niche population. During development, it was recently reported that maternal peripheral inflammation alters fetal ventricular zone proliferation that reflects in cortical layers formation. The effect was shown to involve the barriers of the brain, including the blood-CP-CSF barrier (Stolp et al., 2011). While several reports refer to the impact of neuroinflammation in the adult neurogenesis niches, as in multiple sclerosis, Alzheimer's disease and brain injury (Tepavčević, 2011; Wood et al., 2011; L'Episcopo et al., 2012) (Hamilton and Holscher, 2012), the more subtle effects of inflammation-induced alterations in the CP *per se*, and its impact in the adult SVZ, are presently unknown.

A physiological situation where alterations in the CP properties, in the CSF content and in neurogenesis occur is aging. During aging, mainly at the later ages of life, the CP becomes progressively less efficient in protein synthesis and CSF secretion (Redzic et al., 2005). This malfunction in CSF secretion and abnormal removal of CSF toxic compounds, such as the Alzheimer's disease abeta peptide, might contribute to neuropathology (Preston, 2001; Carro et al., 2005). In fact, alterations in the CP-CSF nexus properties might contribute to the alterations observed in the SVZ in rodents during aging and in models and Alzheimer's disease (Jin et al., 2004; Sotthibundhu et al., 2009; Conover and Shook, 2011).

## Concluding remarks

Under basal normal physiological conditions the CP displays the ability to express several genes encoding for proteins known to promote proliferation, differentiation, and survival of neural progenitor cells. These proteins are secreted toward the CSF, which is as a route for the delivery of CP-born proteins/molecules to the SVZ. *Per se* this is physiologically relevant; yet under disruptive conditions that alter regular CP homeostatic balance, the CSF protein content will be modified and this will lately impact on the SVZ. Whether this culminates in disease developing outcomes (such as is the case of glioblastomas), or might function as potential rescue mechanisms for brain parenchyma lesions (such as in stroke, Parkinson's disease or multiple sclerosis), will be a key issue in adult neural stem cell research in the future. In fact, modulating the CP-CSF nexus in pathologies of the central nervous system could become an important aspect in the usage of endogenous/exogenous neural progenitor cells for stem cell based therapies in the brain.

### Conflict of interest statement

The authors declare that the research was conducted in the absence of any commercial or financial relationships that could be construed as a potential conflict of interest.
